# MAPK-targeted therapies in non-gastrointestinal stromal tumor soft tissue sarcomas: current landscape and future directions

**DOI:** 10.3389/fonc.2025.1418537

**Published:** 2025-08-20

**Authors:** Ouissam Al Jarroudi, Khalid El Bairi, Sami Aziz Brahmi, Said Afqir

**Affiliations:** ^1^ Department of Medical Oncology, Mohammed VI University Hospital, Oujda, Morocco; ^2^ Faculty of Medicine and Pharmacy, Mohamed I University, Oujda, Morocco; ^3^ Faculty of Medical Sciences, UM6P Hospitals, Mohammed VI Polytechnic University, Benguerir, Morocco; ^4^ National Community Oncology Dispensing Association, Cazenovia, NY, United States

**Keywords:** soft tissue sarcomas, MAPK pathway, targeted therapy, precision medicine, clinical trials

## Abstract

Non-Gastrointestinal Stromal Tumors (Non-GIST) Soft Tissue Sarcomas (STS) are highly aggressive and challenging diseases with poor prognosis and limited therapeutic options. Molecular profiling is urgently required to gain a deeper understanding of STS pathogenesis and to identify a comprehensive landscape of genomic alterations in order to develop effective targeted therapies. The mitogen-activated protein kinase (MAPK) signaling pathway is a key molecular mechanism involved in sarcoma development. This study aims to conduct a literature review on the involvement of the MAPK cascade in non-GIST STS, with a focus on the role of MAPK inhibitors in the current treatment paradigm for STS. Furthermore, recent data have provided promising preliminary findings regarding the use of new molecular agents targeting the MAPK pathway, either as single therapies or in combination with other drugs. Numerous clinical trials are currently ongoing, and their outcomes are eagerly awaited. Further research is required in both translational and clinical settings to molecularly characterize STS, identify novel causal alterations, accelerate target discovery, and identify potential biomarkers. Moreover, the development of novel nanomaterials provides a promising perspective that may lead to significant advancements in clinical practice.

## Introduction

Non-Gastrointestinal Stromal Tumor Soft Tissue Sarcomas (non-GIST STS) constitute a broadly heterogeneous group of rare malignant mesenchymal tumors that originate from different tissues, including muscle, adipose, bone, and fibrous tissues (1). These aggressive neoplasms present a significant challenge owing to limited therapeutic options (2). There are approximately 100 distinct histological subtypes, each with unique biological behavior and treatment response (3, 4). Common subtypes include liposarcoma (LPS), leiomyosarcoma (LMS), and undifferentiated pleomorphic sarcoma (UPS). Less prevalent subtypes such as angiosarcoma (AS) and malignant peripheral nerve sheath tumor (MPNST) are also recognized (5). Treatment effectiveness varies among sarcoma subtypes owing to distinct oncogenesis mechanisms. Recent advances in molecular diagnostics have enhanced the understanding of sarcoma genetics, enabling the development of more tailored therapies. Currently, driver mutations have been identified in nearly one-third of sarcoma subtypes. For instance, well-differentiated liposarcoma (WDLPS) and dedifferentiated liposarcoma (DDLPS) are characterized by gene amplification of murine double minute-2 (MDM2) and cyclin dependent kinase 4 (CDK4) (6). Overexpression of hepatocyte growth factor receptor (MET) is observed in clear cell sarcoma (CCS). Anaplastic lymphoma kinase (ALK) gene rearrangement occurs in half of inflammatory myofibroblastic tumor (IMT) cases (7), while disruptions in the mammalian target of rapamycin (mTOR) signaling pathway due to tuberous sclerosis complex 1 and 2 (TSC1 and TSC2) gene mutations are common in perivascular epithelioid cell tumors (PEComas) (8). Notably, angiogenesis, which involves the activation of vascular endothelial growth factor receptors (VEGFR-1 to VEGFR-3), platelet-derived growth factor receptors (PDGFRA and PDGFRB), and other targets, is a common pathway for disease progression in certain sarcoma subtypes. Antiangiogenic drugs (such as pazopanib and regorafenib) have shown effectiveness in common subtypes, such as LMS and synovial sarcoma (SS), but not in lipomatous tumors. These drugs exhibit activity in several rare sarcoma subtypes that are resistant to chemotherapy, such as alveolar soft-part sarcoma (ASPS) (9) or solitary fibrous tumors (SFTs) (10).

A comprehensive, interdisciplinary management strategy is essential for addressing non-GIST STS, as conventional treatments such as surgery, radiation, and chemotherapy have not improved overall survival rates (11, 12). The past two decades have witnessed a significant transformation in GIST management. Extensive research has substantially advanced the understanding of GIST’s pathogenesis and biology. The discovery of the c-KIT mutation was a crucial development, enabling enhanced characterization and identification of GIST through molecular studies. The introduction of imatinib, which selectively inhibits KIT protein tyrosine kinase, has markedly impacted GIST treatment approaches (13). For advanced GIST cases, newly developed TKIs have considerably improved the PFS and OS rates. However, the treatment options for STS remain largely unchanged (14).

The poor prognosis and limited effective therapies for non-GIST STS, particularly in the advanced or metastatic stages, underscore the urgent need to identify targetable molecular alterations and develop novel therapies (15). The integration of advanced molecular techniques into clinical practice has significantly enhanced STS subtyping and treatment options (16). Molecular profiling aims to shift away from a one-treatment-fits-all approach towards more efficacious treatments specific to each non-GIST STS subgroup. Given the high heterogeneity of non-GIST STS, a comprehensive analysis of genetic and molecular profiles utilizing genome- and RNA-sequencing is essential for suggesting molecule-based personalized therapy and improving prognosis (17, 18). To date, the most promising treatment for non-GIST STS involves a molecularly targeted approach that requires elucidation of key molecular mechanisms associated with sarcomagenesis as potential therapeutic targets (19). Furthermore, patients with advanced STS should be encouraged to participate in clinical trials, when available.

The mitogen-activated protein kinase (MAPK) signaling pathway is crucial for the proliferation, migration, and metastasis of STS tumor cells (20). The MAPK cascades facilitate signal transduction through the sequential activation of three to five layers of protein kinases, designated as MAPK kinase kinase kinase (MAPKKKK), MAPK kinase kinase (MAPKKK), MAPK kinase (MAPKK), MAPK, and MAPK-activated protein kinases (MAPKAPK). The initial three central layers constitute a fundamental core unit essential for cell differentiation, proliferation, survival, and angiogenesis. Notably, this pathway is frequently overactive in several malignant tumors, including STS (21). Therefore, inhibition of the MAPK pathway is an important approach for managing non-GIST STS. In this narrative review, we conducted a comprehensive study on the involvement of the MAPK signaling pathway in non-GIST STS and outlined the current state of applying MAPK inhibitors as a potential therapeutic strategy in non-GIST STS.

## MAPK signaling pathway: overview and regulatory mechanisms

**Figure 1:**
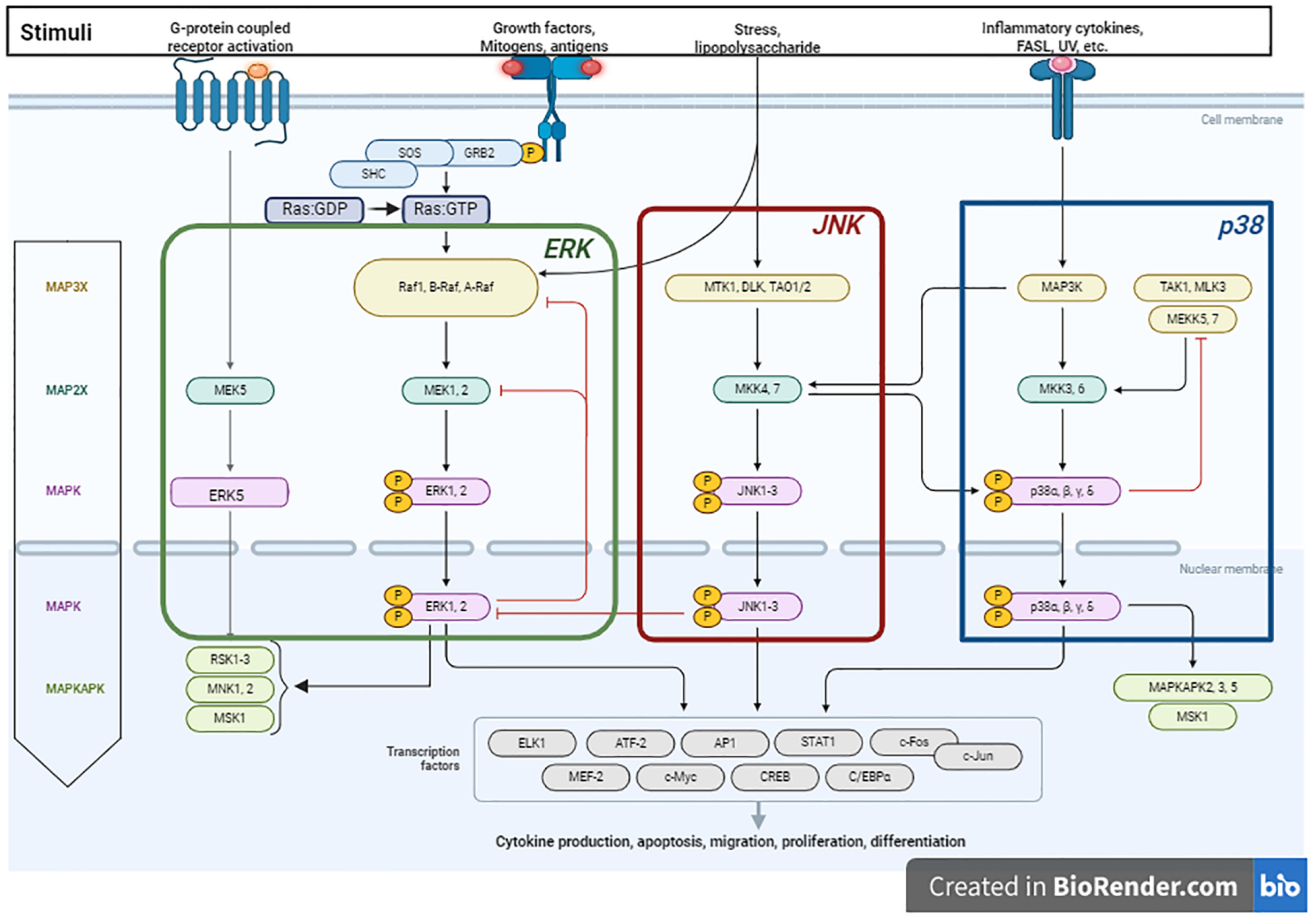
Schematic illustration of the MAPK pathway and its role in promoting tumorigenesis, proliferation and migration. The process is initiated upon the activation of receptor tyrosine kinases (RTKs) by stimuli such as growth factors, cytokines, or stress. The activation of RTKs subsequently leads to the recruitment of the adapter protein Grb2 and the guanine-nucleotide exchange factor Son of Sevenless (SOS), which convert Ras: GDP to Ras: GTP. In mammals, the MAPK pathway comprises three major modules: Extracellular signal-regulated kinase (ERK), C-Jun N-terminal kinase (JNK), and p38 MAPK. The ERK1/2 cascade is activated through the sequential phosphorylation and activation of a series of protein kinases (Raf – MEK1/MEK2 - ERK1/ERK2). RAF kinases, which serve as the MAPKKKK in the ERK1/2 pathway, consist of three members (A-Raf, B-Raf and C-Raf or Raf1). Once activated, ERK translocates to the nucleus, where it activates transcription factors, promoting tumor growth and differentiation by modulating gene expression. The JNK cascade is activated via the phosphorylation of MKK4 and MKK7, which are in turn activated by a variety of MAPKKKs (DLK, MTK1, and TAO1/2). The p38-MAPK module is primarily activated by the protein kinases MKK3 and MKK6. p38 activation extends MAPK cascades by phosphorylating MAPKAPK family members. Figure is adapted from “MAPK Signaling Pathway”, by BioRender.com (2024). Retrieved from https://app.biorender.com/biorender-templates.

The MAPK signaling pathway is a complex network of cellular signal transduction pathways that has been extensively studied in eukaryotic biology, particularly in budding yeast. Early research in this area revealed mechanisms that activate the MAPK pathway and regulate downstream processes (22). The MAPK signaling pathway regulates cellular processes including proliferation, immune responses, and apoptosis. MAPK activation occurs through phosphorylation of substrates in the cytosol and nucleus, modifying protein function and gene expression (23). Activation of the MAPK signaling pathway can be triggered by various factors, such as changes in Ca^2+^ levels, RAS activation, and PKC-mediated or G protein-coupled receptors, in a complex, multistep process involving protein kinase cascades (24) (**Figure 1** **).** Over a dozen MAPK enzymes regulate cell growth, survival, and differentiation (25). Researchers have identified two types of MAPK enzymes: conventional MAPKs and atypical MAPKs (26, 27). Conventional MAPKs include three families of sequentially activated kinases: classical MAPK or ERK (extracellular signal-related kinase), C-Jun N-terminal kinase/stress-activated protein kinase (JNK/SAPK), and p38 MAPK (28). Each group of conventional MAPKs consists of three kinases (MAPK, MAPKK, and MAPKKK) acting sequentially. MAPKKK activation stimulates MAPKK phosphorylation and activation, ultimately triggering MAPK activation. Mammalian cells contain approximately 14 MAPKKKs, 7 MAPKKs, and 12 MAPKs, including ERK1, ERK2, p38α, p38β, p38γ, p38δ, JNK1, JNK2, JNK3, ERK3, ERK4, and ERK5 (24). Conversely, atypical MAPKs form a single group with glycine or glutamine acids, replacing the tyrosine residues in ERK3/ERK4 and NLR (29). The regulatory mechanisms and physiological functions of atypical MAPKs are poorly understood (30).

## Insights into the role of the MAPK signaling pathway in non-GIST STS

The multidisciplinary management of STS, including surgery, radiation therapy, and chemotherapy, has shown limited antitumor efficacy and short survival rates (31). Current research focuses on analyzing diverse signaling pathways in STS to determine their influence on patient outcomes and to identify potential therapeutic targets (20). The MAPK signaling pathway has emerged as a promising target for specific molecular-directed therapy for non-GIST STS (19). Previous preclinical studies have established a strong correlation between the MAPK signaling pathway and cell proliferation in sarcomas, emphasizing the potential therapeutic advantages of selective MAPK inhibitors for treating bone and STS (5). An additional study analyzed the prognostic relevance of MAPK pathway hyperactivation in STS. High expression of phospho-ERK1/2 is associated with aggressive behavior in UPS (32). *In vitro* studies of bone sarcomas have demonstrated the involvement of the MAPK pathway in modulating their behavior by enhancing aggressive properties, such as proliferation, angiogenesis, and inflammation (33). A recent study showed high intrinsic heterogeneity among STS subtypes; however, the MAPK signaling pathway consistently mediates stimuli in STS, regardless of the specific subtype (34). These findings have led to the emergence of MAPK targeting as a potential treatment modality for sarcomas.

## Inhibition of the MAPK pathway: implications for current and future therapy

**Figure 2:**
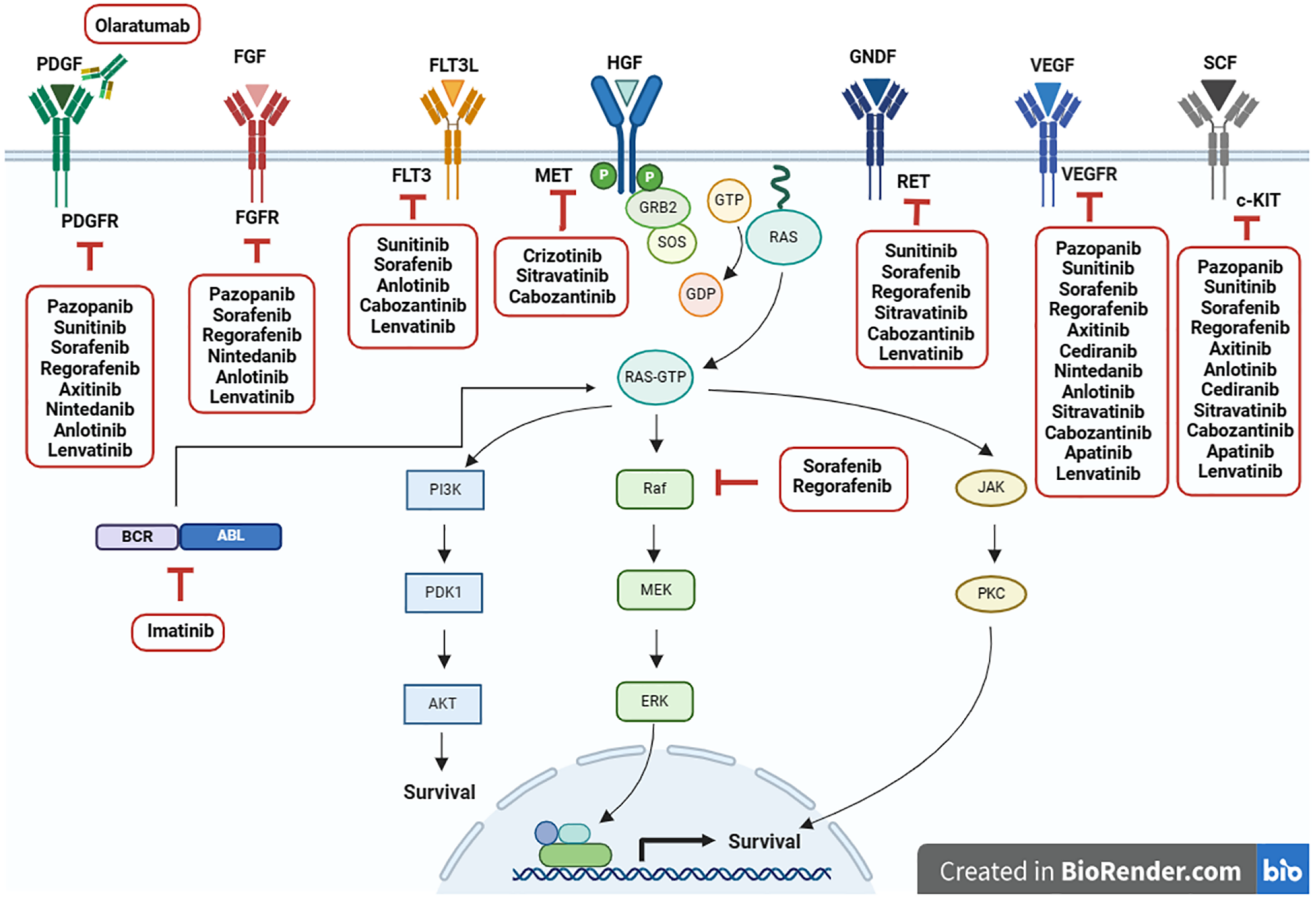
Current targeted therapies for inhibiting MAPK signaling pathway in non-GIST STS. Adapted from “Ras Pathway”, by BioRender.com (2024). Retrieved from https://app.biorender.com/biorender-templates.

Recent studies have highlighted the role of MAPK pathway in STS pathophysiology and its potential as a therapeutic target. Several MAPK-targeted inhibitors are currently available, and additional compounds are being investigated in preclinical and clinical trials (35). Furthermore, crosstalk exists between the AKT/mTOR and MAPK pathways, with RAS activating the RAS/MEK/ERK and PI3K/AKT/mTOR pathways. Mutations in several STS subtypes activate pro-survival and growth factor signaling cascades, thereby promoting sarcomagenesis through downstream pathways (36). Growth factors such as IGF, c-MET, VEGF, and PDGF contribute to STS pathogenesis via the RAS/MEK/ERK and/or PI3K/AKT/mTOR pathways (19). This summary provides an overview of relevant treatments targeting the MAPK signaling pathway in non-GIST STS management (**Figure 2**).

### Pazopanib

Pazopanib is a targeted therapy that specifically inhibits several growth factor receptors, including VEGFR, FGFR, PDGFR, and c-KIT (37). Preclinical studies show its antitumor effects are attributed to inhibiting angiogenesis and inducing apoptosis. Pazopanib also functions as a pan-RAF inhibitor, blocking the MAPK pathway in cancer cells (38). In a phase II clinical trial (EORTC 62043, NCT00297258), pazopanib exhibited safety and potential antitumor activity in advanced STS, except for the adipocytic subtype (39). However, a phase III trial (PALETTE, NCT00753688) found that pazopanib prolonged progression-free survival (PFS) in previously treated non-adipocytic STS patients, without significant improvement in overall survival (OS) (40). This trial resulted in the approval of pazopanib for STS treatment, excluding LPS (41, 42). Given the overall modest activity of pazopanib as a single agent in STS with varying efficacy across histotypes, further research is needed to identify biomarkers for patient selection and to elucidate resistance mechanisms (43). Several combinations of pazopanib with other drugs, such as chemotherapy (NCT01593748) (44) or a PDL1 inhibitor (NCT03798106) (45), have been investigated in phase II clinical trials to improve the incremental activity of pazopanib as monotherapy (46, 47). Two ongoing clinical trials have assessed pazopanib-based combinations. The first trial was a randomized, multi-center phase II study (NCT05679921) that explored the combination of pazopanib with a PD-1 inhibitor (pembrolizumab) in metastatic STS. The rationale for this combination is based on the potentiating antitumor effect of combining antiangiogenic tyrosine kinase inhibitors (TKIs) with immune checkpoint inhibitors (ICI) (48). The second trial was a phase I/II study (AMPHISARC, NCT05180695) with two steps (dose-escalation and dose-extension) that analyzes the potential of combining pazopanib with an MDM2 inhibitor (HDM201) in advanced/metastatic STS with p53-wild type. The aim of this combination is to enhance the anti-growth effect by blocking angiogenesis and inhibiting p53-MDM2 interaction, thereby increasing p53 tumor suppressive activity (49).

### Imatinib

Imatinib is a specific BCR-ABL inhibitor that blocks BCR-ABL-dependent signaling pathways such as the p38 MAPK cascade (50). Following the significant breakthrough of imatinib as the first TKI approved for GIST management, it was subsequently assessed in bone and STS (51). Preclinical data demonstrated the promising efficacy of imatinib in MPNST (52), malignant rhabdoid tumor (MRT) (53), LMS (54), giant cell fibroblastoma and dermatofibrosarcoma protuberans (DFSP) cell lines (55). Its antitumor effect was attributed to reduced cell proliferation and induced apoptosis in both *in vitro* and *in vivo* models (56). In an open-label, single-arm, phase II trial (NCT00031915), imatinib was evaluated in pretreated patients with metastatic and locally advanced sarcomas. This study analyzed 10 subtypes of bone and STS (LMS, LPS, SS, MPNST, fibrosarcoma, rhabdomyosarcoma (RMS), malignant fibrous histiocytoma, AS, osteosarcoma, and Ewing’s sarcoma) (51). The primary endpoint was a clinical benefit rate (CBR) of > 30% for each histotype. However, this objective was not met, indicating a lack of imatinib activity in these selected sarcoma subtypes. Subsequently, an unplanned cohort of desmoid tumors (DT) was embedded in this trial, with promising outcomes: a 58% PFS rate and 84% of patients experiencing stable disease (57). A single-arm phase II study (NCT00287846) dedicated to DT involved 40 patients with progressive disease who received imatinib. The one-year PFS rate was 67%, and 10% of patients discontinued treatment due to toxicities (58). Another phase II trial (NCT01137916) enrolled 38 patients with progressive DT, achieving a one-year PFS rate of 59% (59). Imatinib was also evaluated in DFSP. Two open-label, single-arm, single-agent phase II trials (EORTC-62027, NCT00085475) and (SWOG-S0245, NCT00084630) were conducted. The latter failed to reach its target enrollment of 40 patients due to slow accrual, resulting in termination. Pooled data analysis demonstrated that imatinib is a promising therapeutic option for inoperable DFSP (60). In conclusion, imatinib has shown antitumor efficacy in progressive DT and unresectable DFSP (61).

### Sunitinib

Sunitinib is a multi-target TKI that blocks several growth factor receptors such as VEGFRs, PDGFR, FLT3, RET, CSFR, and c-KIT (62). Mechanistically, its activity is explained by its ability to arrest cell growth resulting from the blockade of ERK, JNK, and p38 MAPK cascades (63). Preclinical studies have shown potential efficacy in selected sarcoma subtypes such as MPNST, MRT, and LMS (64). Various clinical trials have evaluated the effects of sunitinib in bone and STS. In a phase II trial, 53 adult patients with advanced non-GIST STS received sunitinib, which resulted in a median PFS of 1.8 months. After 24 weeks of treatment, 14 patients had disease stability, mirroring the outcomes observed in the placebo arm of the PALETTE phase II trial. This underscores the need to further define the role of sunitinib in STS. In a subsequent phase II trial involving 19 patients with unresectable DT, sunitinib demonstrated antitumor activity. Five patients experienced partial disease, while eight patients had stable disease. However, serious toxicities were observed, likely because of the high prevalence of mesenteric DT (63.2%) (65). Furthermore, retrospective case series have suggested the utility of sunitinib in specific subtypes of non-GIST STS, including ASPS (66), extraskeletal myxoid chondrosarcoma (67), and SFT (68). Although the small number of patients and non-randomized design in these studies limit the ability to draw firm conclusions about sunitinib’s effectiveness in non-GIST STS, it may be considered as a salvage therapy for specific challenging subtypes, which are known for their indolent nature and chemoresistance (61). Combination strategies represent an interesting path for STS management. Sunitinib was explored in combination with nivolumab in a single-arm, phase Ib/II trial (IMMUNOSARC, NCT03277924), which appears to be an active and safe regimen for patients with advanced STS (69).

### Sorafenib

Sorafenib is a specific multitarget inhibitor that selectively inhibits Raf-1, B-Raf, PDGFRβ, VEGFR2, FLT3, RET, and c-KIT (70). In preclinical models, sorafenib has demonstrated antiproliferative activity in DT (71), MPNST (72), and RMS cell lines (73) by blocking ERK, MEK, and AKT signaling cascades (74, 75). The efficacy of sorafenib has been evaluated in clinical trials for various STS, primarily in vascular STS. A phase II single-arm trial (NCT00874874) tested sorafenib in AS, with disappointing results. The median PFS was 1.8 months in the superficial AS group and 3.8 months in the visceral AS cohort (76). A subgroup analysis of patients with progressive SFT (N = 5) showed that two out of five patients achieved 6-month disease control. However, the small sample size limited conclusive findings regarding the antitumor efficacy of sorafenib in STS. Further studies are needed to evaluate the role of sorafenib in this STS subgroup (77). In the same trial, 15 patients with metastatic or inoperable locally advanced epithelioid hemangio-endothelioma (EHE) were evaluated with a response rate of approximately 30.7%. Three patients required a dose reduction, and five patients discontinued the drug (78). A retrospective study of 26 patients with aggressive DT evaluated the sorafenib’s efficacy, showing that 25% of patients experienced partial responses and 70% had disease stability (79). In a randomized, double-blind, placebo-controlled phase III trial (ALLIANCE A091105, NCT02066181), sorafenib was compared to placebo in DT or aggressive fibromatosis (AF). The primary endpoint was PFS rate, and 2-year PFS rate was at 81% in the sorafenib arm versus 36% in the placebo arm. However, OS outcomes were not reported (80). To our knowledge, the ALLIANCE A091105 trial is the only phase III study evaluating TKI in DT, highlighting the promising role of sorafenib in this specific sarcoma histotype.

### Regorafenib

Regorafenib is a multi-kinase inhibitor that effectively targets VEGFR, PDGFR, c-KIT, RET, FGFR, and RAF (81). To achieve its antitumor effect, regorafenib induces the activation of MAPK pathways, including the JNK and p38 MAPK signaling cascades (82, 83). Preclinical studies with different STS cell models demonstrated promising activity in LMS (56), MRT (84), and SFT (61). Subsequently, the efficacy of regorafenib was evaluated in a clinical setting. In a phase II, randomized, double-blind trial (REGOSARC, NCT01900743), regorafenib was compared with placebo in adult pretreated advanced STS patients. The results were stratified according to histological subtype (LPS, SS, LMS, and other STS). Regorafenib showed improved PFS in the LMS, SS, and other STS subtypes but not in the LPS cohort (85). An updated analysis of the REGOSARC trial in 2018 confirmed PFS improvement in the regorafenib arm for patients with non-adipocytic STS after a median follow-up of 32.4 months. However, no OS benefit was observed owing to crossover to the regorafenib group once progression was confirmed in the placebo group (86, 87). Another phase II, prospective, non-randomized, single-center trial (NCT02307500) assessed the clinical activity and safety of regorafenib in adult pretreated advanced STS patients. 21 patients were enrolled, with the primary endpoint being the PFS rate at 8 weeks. After a median follow-up of 33.5 months, the PFS rate was 62% (13 out of 21 patients), confirming the incremental clinical activity of regorafenib in advanced non-adipocytic STS (88). Another phase II, randomized, double-blind, multicenter trial (NCT01900743) was designed to compare regorafenib with placebo in adult patients with unresectable advanced or metastatic STS previously treated with chemotherapy and pazopanib. A significant improvement in PFS was observed in the regorafenib group compared to the placebo group, with a median PFS of 2.1 *vs*. 1.1 months (p = 0.0007), highlighting its promising role as salvage therapy for heavily pretreated non-adipocytic STS (89).

Regorafenib showed promise in bone sarcomas, as demonstrated in the REGOBONE trial (NCT02389244), a randomized, controlled phase II study assessing its efficacy and safety in metastatic bone sarcomas (chondromas, chondrosarcomas, osteosarcomas, and Ewing sarcomas). In the osteosarcoma cohort, regorafenib offered a meaningful benefit over placebo in PFS and OS outcomes, with median PFS of 16.4 weeks and median OS of 11.3 months in the regorafenib group, compared to 4.1 weeks and 5.9 months in the placebo group (90). Therefore, regorafenib can be considered an effective treatment option for osteosarcomas and non-adipocytic STS. It is a promising addition to the armamentarium of targeted therapies for STS treatment. It also represents an interesting option for maintenance therapy of bone sarcomas. Two-phase II clinical trials (NCT04698785 and NCT04055220) are currently recruiting patients with bone sarcomas to study the role of regorafenib as a maintenance treatment. To improve STS outcomes, combinations targeting the VEGFR and PD1/PDL1 pathways have been explored. A recent phase II basket trial (NCT03475953) evaluated the regorafenib-avelumab combination in different solid tumors, including STS (91). Based on these data, further larger studies are required to investigate the best partner to combine with regorafenib and identify potential biomarkers for better patient selection (92). Another phase Ib trial (NCT03475953) is ongoing to further assess the regorafenib-avelumab combination in STS with an immune signature (Tertiary Lymphoid Structure Signature +) (**Table 1**).

**Table 1:** Ongoing clinical trials assessing regorafenib in non-GIST STS.

NCT	Study design	Tumor type	Treatment regimen	N	Primary endpoint	Status	Estimated completion study date
NCT04698785	Randomized, placebo-controlled, double-blinded, phase II study	Patients with high-grade bone sarcomas at diagnosis or relapse and without complete remission after standard treatment	Maintenance treatment by regorafenib and best supportive care (BSC) versus placebo and BSC	60	Progression free survival	Recruiting	July 2026
NCT04055220	Randomized, placebo-controlled, double-blinded, multi-center and 2-arms study	Patients with bone sarcoma after the first line therapy (osteosarcoma, Ewing sarcoma, chondrosarcoma, undifferentiated pleomorphic sarcoma, leiomyosarcoma, angiosarcoma)	Regorafenib as maintenance therapy versus placebo	168	Relapse free survival	Recruiting	October 2026
NCT02389244	Randomized, phase II, placebo-controlled, multi-center study	Metastatic bone sarcomas (conventional high-grade osteosarcoma, Ewing sarcoma of bone, intermediate or high-grade chondrosarcomas and chordomas and either bone or soft tissue metastatic CIC-rearranged sarcomas)	Regorafenib versus placebo	132	Non-progression rate	Recruiting	March 2026
NCT05395741	Open label, randomized and phase I/II	Patients with refractory primary bone tumors	Regorafenib	30	Event free survival	Recruiting	December 2025
NCT05830084	Open label and phase Ib trial	Treatment of newly diagnosed patients with multi-metastatic Ewing sarcoma	Combination of regorafenib with conventional chemotherapy	24	Dose limiting toxicities	Recruiting	March 2026
NCT03475953	Multicenter, prospective, open label, phase Ib trial based on a dose escalation study design (3 + 3 traditional design), followed by independent phase II trials	Patients with advanced digestive solid tumors. Multiple cohorts including STS and solid tumors with immune signature (TLS+).	- Phase I: regorafenib (3 dose levels) given in combination with avelumab (no dose escalation for avelumab) - Phase II: regorafenib at the RP2D associated to avelumab	747	- Phase I: recommended phase II dose (RP2D) - Phase II: antitumor activity of regorafenib	Recruiting	December 2025
NCT04803877	Single-arm, Simon two-stage, historically controlled, phase II study (SARC038)	Patients with refractory or recurrent osteosarcoma	Regorafenib in combination with nivolumab	48	4-month progression-free survival rate	Active, not recruiting	June 2026

### Axitinib

Axitinib is a small-molecule TKI that selectively targets VEGFR, PDGFR, and c-KIT (93). It inhibits tumor growth by blocking VEGFR2, AKT, and ERK signaling pathways (94). Preclinical studies have shown that axitinib is effective in myxoid LPS cell lines and xenografts (95), MRT, SS, and LMS models (56). In a prospective, open-label, non-randomized phase II trial (NCT02261207) involving 17 adult patients with progressive advanced SFT, a partial response was observed in seven patients (7/17, 41%) and stable disease in six patients, while four patients experienced progression. Among the four patients with high-grade or dedifferentiated SFT, no response was reported with axitinib. Furthermore, seven of the 17 patients included in this trial had previously received pazopanib, with half of them responding to axitinib (96). Based on these findings, axitinib may be considered an interesting therapeutic option for advanced SFT after progression on pazopanib. A recent multicenter, open-label, non-randomized, histologically stratified phase II study (Axi-STS, ISRCTN 60791336) of 145 patients with advanced STS found that axitinib demonstrated clinical activity in four histological strata (AS, LMS, SS, and other non-adipocytic STS), with further confirmation needed in phase III trials (97). The combination of VEGFR and ICI is being explored due to the role of angiogenesis in sarcoma proliferation and immunosuppression. In a phase II trial (NCT02636725), the axitinib-pembrolizumab combination was assessed in 36 patients with advanced or metastatic STS, showing meaningful clinical activity, with a 3-month PFS rate of 65.6% in all patients and 72.7% in the ASPS cohort (98).

### Cediranib

Cediranib is a TKI that inhibits VEGFR and c-KIT (99), causing antiproliferative effects by suppressing the VEGFR, AKT/mTOR, and ERK/MAPK signaling cascades (100). Tumor regression has been observed in specific rhabdoid tumor xenograft models (101) and MRT, SS, and LMS cell lines (56). Cediranib has demonstrated preliminary activity in ASPS patients (35). In a single-arm phase II study (NCT00942877), cediranib was investigated in 46 adult patients with unresectable or metastatic ASPS. Radiological response was assessed using the RECIST criteria. Partial response and stable disease were reported in 15 (35%) and 26 (60%) patients, respectively, providing evidence of cediranib activity in ASPS (102). In a phase II trial (CASPS, NCT01337401), cediranib was compared with placebo in metastatic ASPS patients. An improvement in response rate was reported in the cediranib arm versus the placebo group (19% *vs*. 0%, p = 0.072). However, no significant benefit was observed between the two cohorts (p = 0.28) (103). The data suggest a preliminary activity of cediranib in ASPS (104), but larger cohorts are needed to confirm these results and understand the resistance mechanisms.

### Nintedanib

Nintedanib is a TKI targeting VEGFR, PDGFR, and FGFR (105), with antitumor effects mainly attributed to the dual inhibition of the ERK and AKT signaling pathways (106). It showed preclinical activity in MPNST (107) and SS models (108), leading to further clinical development. However, a phase II trial (EORTC1506, NCT02808247) comparing nintedanib with ifosfamide in second-line STS treatment was halted due to futility. The trial failed to provide evidence supporting the clinical use of nintedanib in advanced, unselected STS (109).

### Anlotinib

Anlotinib is a selective TKI that inhibits VEGFR, PDGFR, FGFR, FLT3, and c-KIT (110). Its antitumor effect is attributed to blockade of the ERK/MAPK and PI3K/AKT signaling pathways (111, 112). In preclinical settings, anlotinib has shown activity in SS models (113). In the phase II single-arm trial (NCT01878448), anlotinib was evaluated as a second-line treatment after progression on anthracycline therapy for 166 patients with advanced STS. They achieved a 74% disease control rate with median PFS and median OS of 5.6 and 10.7 months, respectively (114). This trial suggests a meaningful activity of anlotinib in STS (115). In a phase II/III clinical study comparing the efficacy and safety of anlotinib with a placebo in 233 patients with STS (ALTN-02-IIB, NCT02449343), anlotinib was identified as a novel treatment option for patients with advanced STS after the failure of standard chemotherapy (116). A subsequent phase III trial (APROMISS, NCT03016819) confirmed the acceptable benefit-risk profile of anlotinib in patients with advanced SS, demonstrating improved disease control and superior PFS compared with dacarbazine in advanced SS (117).

Anlotinib has also been utilized as a first-line treatment for locally advanced or metastatic STS in patients unfit for chemotherapy, exhibiting encouraging anti-tumor activity and favorable tolerability in a Chinese phase II clinical trial (NCT03792542) (118). Further studies are necessary to identify the most beneficial STS subgroup. Based on these preliminary promising results, anlotinib is currently being investigated in advanced ASPS, LMS, and SS in an ongoing phase III trial (APROMISS, NCT03016819) (117). Additionally, anlotinib was combined with irinotecan for advanced Ewing sarcoma after standard therapy failure in a multicenter, single-arm phase Ib/II trial (NCT03416517), demonstrating promising clinical efficacy (119). An open-label and single-arm phase II trial (ALTER-S006, NCT03890068) assessed its efficacy as a maintenance therapy in 49 patients with advanced STS who achieved stability after first-line chemotherapy (anthracycline-based treatment). It showed meaningful activity as a post-chemotherapy maintenance treatment in advanced STS, with a median PFS of 9.1 months (120). In a double-blind phase II clinical trial (NCT03951571), the role of anlotinib as an adjuvant therapy for completely resected high-grade STS was evaluated, suggesting its impact in reducing disease recurrence risk with an acceptable toxicity profile. Anlotinib is currently being studied in combination with other therapies in retrospective real-world studies. The anlotinib-liposomal doxorubicin combination was evaluated in an observational study that analyzed the efficacy and safety of this combination in 27 patients with metastatic STS. This Chinese study highlighted the efficacy of anlotinib-liposomal doxorubicin followed by anlotinib monotherapy in advanced STS (121). Another study found that anlotinib was associated with PD-L1 inhibitors in a retrospective cohort of 32 patients with pretreated metastatic STS, achieving an ORR of 34.4% and median PFS of 7.6 months. This study provides real-world evidence of the efficacy of anlotinib-based combinations in advanced STS (122). However, further prospective clinical trials are needed to identify the optimal partner of anlotinib in STS management. Several anlotinib-based combinations are ongoing, including chemotherapy (NCT05121350, NCT05747521), anti-CD137 (NCT05301764), and ICI (NCT04172805, NCT05481645, NCT05193188, and NCT05926700) (**Table 2**).

**Table 2:** Ongoing clinical trials investigating anlotinib in non-GIST STS.

NCT	Study design	Tumor type	Treatment regimen	N	Primary endpoint	Status	Estimated completion study date
NCT03890068	Single-arm, multi-center, phase II trial (ALTER-S006)	Advanced STS	Anlotinib hydrochloride maintenance treatment after first-line anthracycline-based chemotherapy	48	Progression free survival	Recruiting	May 2024
NCT05121350	Multicenter, randomized, double-blind, parallel-controlled phase III trial	First-line treatment of advanced STS	Anlotinib hydrochloride combined with epirubicin hydrochloride versus placebo combined with epirubicin hydrochloride	256	Progression free survival	Recruiting	June 2024
NCT05747521	Single-arm, single-center, prospective investigator-initiated clinical study	High-grade STS	Anlotinib hydrochloride combined with doxorubicin and radiotherapy	58	Objective response rate	Recruiting	September 2024
NCT05602415	Open label, phase II study	Resectable STS with high recurrence risk	Postoperative radiotherapy with tyrosine kinase inhibitor (anlotinib)	41	Local recurrence free survival	Recruiting	May 2024
NCT05481645	Multi-center, open label, randomized, controlled, phase II clinical trial	First-line treatment of patient with advanced endometrial cancer or sarcoma of uterus	TQB2450 injection (PDL-1 inhibitor) combined with chemotherapy ± anlotinib hydrochloride	79	Investigator-assessed objective response rate	Recruiting	December 2024
NCT04172805	Single-arm, open label, phase II study	Refractory and advanced STS	Anlotinib combined with toripalimab (PD-1 inhibitor)	70	Objective response rate	Recruiting	May 2024
NCT05193188	Open label, randomized, multicenter clinical controlled phase II study	Unresectable high-grade chondrosarcoma with different IDH genotypes	Anlotinib combined with PD-1 antibody	70	6 month-progression free survival rate	Recruiting	March 2026
NCT05926700	Open label, single-arm, phase II trial	Advanced or metastatic STS with previous first-line standard treatment failure	Cadonilimab (PD-1 and CTLA-4 inhibitor) combined with anlotinib	27	Objective response rate	Not yet recruiting	January 2025
NCT05301764	Open label, phase Ib/II trial	Locally advanced, metastatic or recurrent refractory STS	LVGN6051 (anti-CD137) combined with anlotinib	65	- Dose limiting toxicities - Safety - Objective response rate	Recruiting	October 2025

Overall, anlotinib demonstrates potential as a treatment for advanced sarcomas, functioning as an anti-angiogenesis TKI with significant effects, manageable adverse reactions, and enhanced efficacy in combination therapies. However, several challenges persist, including drug resistance, determination of optimal dosage, combining with conventional anti-cancer medications, sequencing, and assessing of treatment effectiveness. To achieve optimal outcomes utilizing anlotinib as targeted therapy for advanced sarcoma patients, these issues warrant investigation for individual sarcoma types (115).

### Sitravatinib

Sitravatinib inhibits VEGFR, c-KIT, RET, and MET (123). In preclinical models, sitravatinib induced growth inhibition in DDLPS and MPNST cell models by deactivating the PI3K/AKT and RAS/MAPK pathways (124). This preclinical activity has translated into clinical settings, leading to an ongoing single-arm phase II trial (NCT02978859) in advanced LPS and other STS (125). The trial evaluated patients with advanced LPS (WDLPS and DDLPS) who received at least one systemic therapy in the metastatic setting. The primary endpoint was PFS rate at 12 weeks. 12 of the 29 patients included had free progression at 12 weeks, suggesting the potent efficacy of sitravatinib in advanced LPS (126).

### Crizotinib

Crizotinib is a small-molecule TKI that targets the ALK and MET signaling cascades (127), demonstrating antitumor effects in small round-cell tumors and SS models (128). It inhibits cell proliferation by blocking the ERK, AKT, and STAT3 pathways (129). The CREATE trial (EORTC90101, NCT01524926) is a non-randomized, single-arm phase II study that investigated the efficacy of crizotinib in IMT, ASPS, CCS, and aRMS (130, 131). This evaluation is based on ALK and/or MET activation in the pathogenesis of these subgroups (132, 133). In this trial, a cohort of 48 patients with unresectable advanced or metastatic ASPS was analyzed, with a partial response in two patients and stable disease in 39 patients. Concerning crizotinib safety, grade 3 and 4 adverse events were reported in six out of the 48 patents (12.5%) included in the ASPS cohort. In the IMT cohort, crizotinib showed a 50% ORR in patients with ALK gene rearrangement. The updated results in 2021 showed a 66.7% ORR in ALK-positive IMT and a 14.3% ORR for ALK-negative IMT. These findings confirmed that crizotinib is an effective therapy for advanced ALK-positive IMT (134).

CCS are rare tumors characterized by a pathognomonic translocation of t (12,22)(q13; q12), leading to an EWSR1/ATF1 gene fusion (135, 136). The ESWR1/ATF1 transcriptional activator is regulated by the upstream p38 MAPK signaling pathway (137, 138). In the CCS cohort of the CREATE trial, 34 patients were enrolled. Only one patient showed a partial response, while 17 patients had stable disease. The median PFS reported with crizotinib (4.4 months) was higher than that observed in CCS patients treated with cytotoxic chemotherapy in the metastatic setting (131, 139). CREATE is the first genotype-driven trial to evaluate TKI activity in non-GIST STS patients. These findings underscore the importance of screening for ALK status as a biomarker for crizotinib’s efficacy in ALK-rearranged IMT.

### Dasatinib

Dasatinib is another TKI that targets SFK members and some TK receptors (such as EGFR and ephrin receptors), inhibiting their corresponding downstream signaling pathways (RAS/ERK, STAT3, FAK, and PI3K/AKT) (140). It demonstrated meaningful preclinical activity in various STS subgroup models (aRMS, LPS, SS, ASPS, and MRT) (56, 141–143). However, it failed to show clinical activity in a phase II trial (SARC009, NCT00464620) for advanced STS patients with SFT, ASPS, epithelioid sarcomas, chondrosarcomas, and chondromas (144).

### Cabozantinib

Cabozantinib is an interesting multi-target TKI that inhibits umpteen TK receptors (VEGFR, FLT3, AXL, RET, c-KIT, and MET) (145–147). These targets are involved in the pathogenesis of sarcomas. In preclinical studies, cabozantinib showed significant activity in ASPS and RMS cell lines (143, 148). Additionally, it has been demonstrated to inhibit cell growth in osteosarcomas and Ewing sarcomas in preclinical models (149, 150). In the clinical setting, after demonstrating potent efficacy in GIST, cabozantinib was further investigated in bone and STS in a phase II clinical trial (NCT02216578) (151). Subsequently, it was investigated in a phase I trial (NCT01709435) for refractory solid tumors, including six patients with STS (ASPS, CCS, RMS, and SS) and six patients with bone sarcomas (osteosarcomas and Ewing sarcomas), leading to further research on cabozantinib in bone and STS (152). A phase II study (NCT01755195) was designed to assess the efficacy and safety of cabozantinib in 54 patients with advanced and previously treated STS. The study reported an ORR of six patients (11.1%) and a 6-month PFS rate of 49.3%. Cabozantinib shows promise as a therapy for selected STS subtypes (ASPS, LMS, UPS, and extra-skeletal myxoid chondrosarcoma).

The CABONE trial (NCT02243605) was a phase II study that enrolled 90 patients with heavily pretreated osteosarcomas (N = 45) and Ewing sarcomas (N = 45). The primary endpoints were ORR and 6-month PFS. In the osteosarcoma cohort, the ORR was 12% with a median PFS and median OS of 6.7 and 10.6 months, respectively. In the Ewing sarcoma cohort, the ORR was 26%, with median PFS of 4.4 months and median OS of 10.2 months. Cabozantinib caused serious adverse events in 68% of patients, but no grade 5 toxicity was reported (153, 154). In a recent phase II trial (NCT02867592), the activity of cabozantinib was analyzed in children and young adults with refractory solid tumors, including osteosarcomas, Ewing sarcomas, RMS, and non-RMS STS. This trial found promising activity of cabozantinib in osteosarcoma patients, with an ORR of 34% (155). The combination of cabozantinib with other therapies was also evaluated in heavily pretreated LMS patients, showing an increase in CBR of 33% (156). The cabozantinib-nivolumab combination was assessed in patients with advanced or metastatic AS who had previously received taxane chemotherapy in a phase II trial (Alliance A091902, NCT04339738). The study found a preliminary antitumor effect in patients with advanced taxane-pretreated AS. Exploratory analyses are still awaited (157). Cabozantinib-based combination with dual ICI (PD-1 and CTLA-4 inhibitors) was investigated in a phase II trial (NCT04551430) in metastatic STS. The study aimed to compare two arms: cabozantinib as monotherapy and cabozantinib in association with ipilimumab and nivolumab for 4 cycles, followed by nivolumab as maintenance. The triplet arm had a significantly higher disease control rate (DCR) than the single-agent arm (80% *vs*. 42%, p = 0.0004). PFS was also improved in the triplet arm compared to the single arm with a median PFS of 5.4 *vs*. 3.8 months, respectively (p = 0.016) (158). Cabozantinib is a potent and effective therapeutic option for bone and STS management. The drug is still being explored in various ongoing clinical trials for patients with STS in different settings. In the neoadjuvant setting, cabozantinib is being analyzed in combination with radiation therapy for extremities sarcomas in a phase I/II trial (NCT04220229). Cabozantinib is being investigated as a maintenance therapy in an ongoing phase II trial (NCT01979393) for patients with high-grade uterine sarcomas (159). Moreover, cabozantinib has been assessed in combination with ICI in three ongoing phase II clinical studies in patients with bone and STS: with PD-1 inhibitor (pembrolizumab, NCT05182164), PDL-1 inhibitor (atezolizumab, NCT05019703), and PD-1/CTLA-4 inhibitors (nivolumab plus ipilimumab, NCT04551430) (**Table 3**).

**Table 3:** Ongoing clinical trials assessing cabozantinib in non-GIST STS.

NCT	Study design	Tumor type	Treatment regimen	N	Primary endpoint	Status	Estimated completion study date
NCT01979393	Randomized, double-blind phase II study	High-grade uterine sarcoma (HGUtS)	Maintenance therapy with cabozantinib after stabilization or response to doxorubicin +/- ifosfamide following surgery or in metastatic first-line treatment	58	Progression free survival	Active, not recruiting	May 2024
NCT04220229	Open label, phase I/II study	Sarcomas of the extremities	Neoadjuvant treatment by cabozantinib in combination with radiation therapy	46	- Recommended phase 2 dose of cabozantinib (Phase I) - Rate of relapse (Phase II)	Recruiting	July 2026
NCT06156410	Open label, single-arm, phase I trial	Adults and children with relapsed/refractory Ewing sarcoma and osteosarcoma	Cabozantinib in combination with high dose ifosfamide	30	Maximum tolerated dose/recommended phase II dose (MTD/RP2D) of cabozantinib	Recruiting	November 2028
NCT05182164	Single-arm, open label, non-randomized, multicenter, phase II trials, based on 2-stage Simon’s optimal design	3 distinct populations of sarcomas: Stratum 1: advanced undifferentiated pleomorphic sarcoma Stratum 2: advanced osteosarcoma Stratum 3: advanced Ewing sarcoma	Association of pembrolizumab and cabozantinib	119	Efficacy (independently for each stratum)	Recruiting	October 2025
NCT05019703	Open label and phase II trial (TACOS)	Adolescents and young adults with recurrent/metastatic osteosarcoma	Atezolizumab and cabozantinib	40	Progression free survival	Recruiting	December 2027
NCT04551430	Randomized phase II Trial	Metastatic STS	Cabozantinib combined with PD-1 (nivolumab) and CTLA-4 (ipilimumab) inhibition	105	Radiographic response rate by RECIST 1.1	Active, not recruiting	August 2028

### Apatinib

Apatinib is a small-molecule TKI that inhibits VEGFR, c-Src, and c-KIT tyrosine kinases (160), resulting in an antitumor effect due to the inhibition of ERK/MAPK and PI3K/AKT signaling cascades (161, 162). Apatinib was explored in a phase II trial (NCT03121846) in metastatic STS patients. The PFS rate at 12 weeks was 70%, with an ORR of 26.3% (163). However, it did not show a significant improvement in survival in patients with LMS compared to other STS subtypes (164). In a phase II clinical trial, the efficacy and safety of apatinib were analyzed in patients with advanced previously treated STS, which showed moderate antitumor activity of apatinib (165). To improve its effectiveness, apatinib-based combinations were explored. A meaningful antitumor effect was reported in patients with refractory osteosarcomas treated with apatinib combined with cytotoxic chemotherapy (ifosfamide and etoposide) in a retrospective study (NCT04690231) (166). Another combination of apatinib was evaluated in a phase II trial (NCT03359018), showing a PFS benefit in patients with advanced osteosarcomas receiving camrelizumab (a PD1 inhibitor) and apatinib compared to apatinib alone (167). There are three ongoing clinical trials assessing apatinib-based combinations, including two with cytotoxics (NCT04012827 and NCT04824352) and one with ICI (NCT04074564) (**Table 4**).

**Table 4:** Ongoing clinical trials evaluating apatinib in non-GIST STS.

NCT	Study design	Tumor type	Treatment regimen	N	Primary endpoint	Status	Estimated completion study date
NCT04072042	Open label, biomarker- driven, phase II trial	Patients with recurrent or refractory advanced bone and STS	Apatinib monotherapy	30	Progression free rate	Recruiting	May 2024
NCT04012827	Single-arm, open label, multicenter phase II study	Advanced STS	Apatinib mesylate combined with doxorubicin and ifosfamide	108	Progression free survival	Recruiting	December 2023
NCT04824352	Prospective, multiple-center, single-arm, open label and phase II trial	Relapsed or refractory osteosarcoma progressed upon first-line chemotherapy	Apatinib in combination with ifosfamide and etoposide (IE)	44	Progression free survival	Recruiting	April 2024
NCT04074564	Exploratory, open label, randomized study	Patients with advanced bone and STS	Multi-Antigen Stimulated Cell Therapy-I Injection (MASCT-I) combined with apatinib mesylate and/or camrelizumab (PD-1 inhibitor)	60	Safety	Recruiting	December 2024
NCT05235100	Open label, phase II trial	Localized extremity or trunk sarcoma	Preoperative Intensity-modulated Radiotherapy (IMRT) with concurrent apatinib mesylate	30	Rate of major wound complications within 4 months post-surgery	Recruiting	December 2024

### Lenvatinib

Lenvatinib is a potent inhibitor of VEGFR, PDGFR, FGFR, RET, FLT3, and c-KIT. It induces an antitumor effect by blocking the MAPK signaling pathway (168). In a phase Ib/II trial (LEADER, NCT03526679), lenvatinib was combined with eribulin in 20 patients with advanced LPS and LMS, achieving an ORR of 27% and a median PFS of 56 weeks, highlighting the potential efficacy of this combination in LMS and LPS (169). In an open-label, single-arm phase II trial (NCT04784247), lenvatinib was combined with ICI (pembrolizumab) in five cohorts of selected metastatic and unresectable bone and solid tumors (A: LMS; B: high-grade UPS; C: vascular sarcomas (including AS and EHE); D: other STS (including SS and MPNST); and E: bone sarcomas (including osteosarcoma and chondrosarcoma)). Preliminary results indicated the potential antitumor activity of SS, MPNST, AS, and osteosarcoma (170). The trial is ongoing and definitive results are awaited.

### Olaratumab

Olaratumab is a monoclonal antibody that inhibits PDGFR alpha, blocking downstream signaling cascades such as the MAPK pathway (171). Inhibition of proliferation was observed with olaratumab in sarcoma cell lines (172). It was first evaluated in two earlier phase I dose-escalation studies in advanced solid tumors, showing preliminary antitumor effects (173, 174). Olaratumab was then investigated in clinical trials as monotherapy and in combination with other agents. In a randomized phase Ib/II trial (NCT01185964), the efficacy and safety of olaratumab and doxorubicin combination compared to doxorubicin alone in 133 patients with advanced STS were evaluated. The median PFS was 6.6 months in the combination arm versus 4.1 months in the doxorubicin monotherapy group. A statistically significant improvement in OS was reported with the olaratumab–doxorubicin combination compared to doxorubicin alone (26.5 *vs*. 14.7 months, respectively; p = 0.0003) (175).

Olaratumab was the first monoclonal antibody approved by the US Food and Drug Administration (FDA) in October 2016 for treating adult patients with advanced STS (176). However, the combination failed to confirm its efficacy and OS improvement in a subsequent phase III trial (ANNOUNCE, NCT02451943), leading to its withdrawal from the market (177, 178). Recently, a randomized, multicenter phase Ib/II study (ANNOUNCE 2, NCT02659020) was designed to investigate the pharmacokinetics, safety, and efficacy of olaratumab combined with gemcitabine and docetaxel in locally advanced or metastatic STS. This trial failed to demonstrate a significant benefit of OS in the combination arm (179). The addition of olaratumab to PD1 or PDL1 inhibitors appeared to have synergistic activity by increasing the immune response. In a phase Ia/Ib clinical trial (NCT03126591), olaratumab-pembrolizumab combination showed a safe profile, but further studies are required to assess its efficacy (180).

## Mechanisms of resistance to MAPK pathway inhibition in non-GIST STS

Resistance to targeted therapies is a prevalent issue in solid tumors, with several overarching mechanisms identified across various anticancer treatments. By examining these resistance mechanisms, the objective is to develop combination therapies that can preemptively address and counteract resistance at the initiation of treatment, thereby enhancing both the extent and duration of clinical efficacy (181). Although distinct resistance mechanisms are reported in STS for each therapy, the most prevalent include drug inactivation or modification, mutation of the target protein, reduced drug accumulation, and bypass of target inhibition (182).

The reactivation of downstream signaling pathways through the development of secondary mutations in the oncogenic target or mechanisms independent of the original drug target leads to the development of acquired resistance. The reactivation of key downstream effectors through parallel signaling pathways of other receptor tyrosine kinases (RTKs) provides an alternate route around the inhibited target to activate downstream signaling and allows the tumor to bypass the inhibition of the driver gene by the TKI drug and continue to survive and grow. This mechanism of resistance is known as bypass activation (183).

Recent data indicate that the tumor microenvironment (TME) remains not sufficiently understood, particularly regarding its role in drug resistance. Consequently, a thorough evaluation of the TME, along with well-designed histotype-specific preclinical studies, may enhance our understanding of the potential effects of combinatorial treatment strategies in STS. This understanding could facilitate the development of therapeutic interventions tailored to patients with STS by inhibiting alternative pathways (184).

Moving forward, there will be an increasing reliance on computational modeling methods capable of analyzing and testing all reasonable drug combinations. These methods should consider and exploit the complex interactions within the MAPK pathways using in silico computational models. Additionally, employing an ex vivo drug sensitivity platform, such as the quadratic phenotypic optimization platform (QPOP), may prove promising in predicting treatment responses and guiding the development of novel therapeutic strategies for STS patients (185).

## Perspectives and emerging therapeutic options targeting the MAPK pathway in non-GIST STS

A landscape of targeted therapies has been developed for STS treatment, with numerous molecularly targeted agents under investigation to enhance efficacy and overcome potential resistance mechanisms. While significant challenges persist, including the heterogeneity of non-GIST STS subtypes and the potential for adaptive resistance, the ongoing development of MAPK pathway-targeted therapies represents a promising avenue for improving outcomes in patients with these challenging malignancies (186) (**Table 5**).

**Table 5:** Ongoing clinical trials investigating the inhibition of MAPK cascade in non-GIST STS.

Drugs	Target	NCT	Study design	Tumor type	Treatment regimen	N	Primary endpoint	Status	Estimated completion study date
Adagrasib	KRAS	NCT06024174	Open label, randomized and phase I/II study	Participants with KRAS G12C-mutant advanced solid tumors	BMS-986466 in combination with adagrasib with or without cetuximab	410	- Dose limiting toxicities - Safety - Objective response rate	Recruiting	July 2029
Avapritinib	PDGFR and c-KIT	NCT04771520	Open label, single-arm and phase II study	Patients with c-KIT or PDGFRA mutation-positive malignant solid tumors	Avapritinib	50	Objective response rate	Recruiting	February 2025
Chiauranib	VEGFR, PDGFR and c-KIT	NCT05497843	Open label, multicenter, phase II study	Advanced or unresectable STS previously failed to standard of care treatment	Chiauranib monotherapy	40	12 weeks - progression free survival	Recruiting	June 2024
Cobimetinib	MEK	NCT04079179	Open label, non-randomized and phase II trial	Refractory langerhans cell histiocytosis, LCH-associated neurodegenerative disease, and other histiocytic disorders	Cobimetinib	90	Objective response rate	Recruiting	December 2029
		NCT04216953	Multicenter, open label, phase I-II study	Pediatric and adult patients with locally advanced and/or metastatic STS	Combination of a MEK inhibitor and a PDL1 inhibitor (cobimetinib and atezolizumab)	320	16 weeks - progression Free rate	Recruiting	February 2027
CPL304110	FGFR	NCT04149691	Phase I, open label, multicenter, dose escalation study, containing 3 parts: -Initial dose escalation (Part 1 - without FGFR molecular aberrations) -Dose escalation (Part 2 - with FGFR molecular aberrations) -Dose extension (Part 3 - with FGFR molecular aberrations).	Adult subjects with advanced solid malignancies	Oral CPL304110	42	- Maximal tolerated dose - Safety profile	Recruiting	June 2024
Navtemadlin	MDM2	NCT03217266	Phase Ib trial	Wild-type P53 STS	Neoadjuvant AMG 232 (KRT-232) concurrent with preoperative radiotherapy	46	Maximum tolerated dose/recommended phase 2 dosage	Active, not yet recruiting	December 2023
Selpercatinib	RET	NCT03899792	Open label and phase I/II study	Pediatric patients with advanced RET-altered solid malignancies including STS or primary central nervous system tumors	Oral RET inhibitor (LOXO 292, selpercatinib)	50	Dose limiting toxicities	Recruiting	May 2029
Sotorasib	KRAS	NCT04185883	Open label and phase Ib/II study	Subjects with advanced solid tumors with KRAS p.G12C mutation	Sotorasib monotherapy and in combination with other anti-cancer therapies (trametinib + panitumumab; RMC-4630; afatinib; panitumumab +/- FOLFIRI; atezolizumab; carboplatin, pemetrexed, docetaxel, paclitaxel, pembrolizumab; palbociclib; pembrolizumab; bevacizumab-awwb + FOLFIRI or FOLFOX; TNO155; BI 1701963; AMG 404; everolimus)	1143	- Phase 1b: Dose limiting toxicities Safety Pharmacokinetics - Phase 2: Objective response rate	Recruiting	October 2027
Surufatinib	VEGFR, FGFR and CSF-1R	NCT05839275	Prospective phase Ib/II trial (IRIS)	High-risk localized STS	Radiotherapy combined with TKI and ICI (surufatinib and sintilimab (anti-PD1))	52	Objective response rate	Recruiting	July 2029
		NCT05106777	Multi-center, open label phase II study	Patients with osteosarcoma and STS	Surufatinib	47	12 weeks – progression free rate	Recruiting	December 2023
		NCT06110650	Single-arm, single-center, exploratory phase II study	Patients with STS who have failed anthracycline-containing chemotherapy and who have been successfully targeted with anti-vascular agent	Surufatinib monotherapy	29	Progression free rate	Not yet recruiting	August 2025
		NCT05722977	Single-arm, phase II Study	Advanced STS	Surufatinib combined with envafolimab (anti-PDL1) followed by surufatinib as second or more-line therapy	45	Objective response rate	Not yet recruiting	February 2027
TURALIO(R)	CSF-1R and c-KIT	NCT02390752	Open label, non-randomized and phase I Trial	Children and young adults with refractory leukemias and refractory solid tumors (including Neurofibromatosis type 1 (NF1) associated, Plexiform Neurofibromas (PN) and Tenosynovial Giant Cell Tumor (TGCT))	Pexidartinib (PLX3397)	54	- Phase I: Determine a phase II dose of TURALIO(R) and evaluate the safety and tolerability of TURALIO(R)	Recruiting	December 2025

### Other VEGFR-associated multi-targeted TKIs

Angiogenesis is crucial in sarcomagenesis, and VEGFR overexpression is linked to poor prognosis and resistance to cytotoxic drugs in STS (187). Multi-targeted TKIs that inhibit VEGFR are being explored in clinical trials for their potential in treating sarcomas. Tivozanib, a VEGFR-targeting TKI, showed promising results in a phase II trial with 58 patients with advanced unresectable or metastatic STS, with a PFS at 16 weeks of 36.4%. Based on these promising findings, further investigation of tivozanib in combination with other agents is warranted to improve the outcomes in patients with refractory advanced non-GIST STS. Fruquintinib, another antiangiogenic TKI, demonstrated significant improvement in PFS in an exploratory, retrospective study of 122 patients with advanced bone and STS. The median PFS was 4.8 months in the fruquintinib group versus 1.4 months in the control group (p < 0.001) in an exploratory, retrospective study (NCT06202599). This study concludes the place of fruquintinib, a potentially effective and safe drug, in third- or further-line therapy for advanced bone and STS, and sheds light on the need to continue the exploration of this TKI in STS. Additionally, two new anti-VEGFR TKIs, chiauranib and sufuratinib, are currently under investigation in ongoing clinical trials. Chiauranib is being assessed in a multicenter, single-arm phase II trial (NCT05497843) for patients with advanced STS who have previously failed standard therapy or have no standard of care. Sufuratinib is being explored in multiple studies as a single agent (NCT05106777 and NCT06110650) or in combination with PD1/PDL1 inhibitors (NCT05839275 and NCT05722977).

### MEK inhibitors

Inhibition of MEK resulted in the prevention of ERK phosphorylation, which halted tumor growth (188). MEK inhibitors are currently being developed and investigated as monotherapies or in combination with other targeted and cytotoxic agents for STS treatment (189). A recent approach involves combining MEK inhibitors with ICI to enhance immune recognition and augment T cell activity against neoplastic cells. The COTESARC trial (NCT04216953), a phase I/II study, is currently evaluating the efficacy of cobimetinib-atezolizumab combination in both adult and pediatric patients with advanced STS (190).

Furthermore, simultaneous inhibition of MEK and RAF kinase offers advantages in terms of increased efficacy and reduced adverse effects, potentially serving as a promising strategy for targeting the MARK pathway (191). Few cases have reported BRAF mutations and outcomes of BRAF-targeted therapy in various sarcoma types (16). A documented case of BRAF V600E-mutated undifferentiated sarcoma demonstrated successful treatment with a combination of BRAF and MEK inhibitors, suggesting that the BRAF V600E mutation could be a viable therapeutic target when addressed with dual BRAF and MEK inhibition (192).

Considering the high frequency of MAPK pathway abnormalities, particularly in MPNST, MEK inhibitor treatment may prove effective based on preclinical data, warranting further evaluation through clinical trials (193). A comprehensive study of multiple FDA-approved and promising MPNST therapies revealed that low-dose MEK inhibitors demonstrate the strongest synergy and efficacy when combined with other agents. This led to the hypothesis that MEK inhibitors may sensitize MPNST cells to other treatments, potentially rendering combination therapy more effective than mono-therapeutic approaches (194).

Recent research on NF1-deficient MPNST indicated that this subtype develops resistance to MEK inhibitor treatment partly by increasing PDGFRβ transcription and RAF dimer formation. A combination of MEK inhibitors and PDGFR/RAF-dimer inhibitors may overcome this resistance, offering a novel targeted therapeutic approach for NF1-deficient MPNST patients (195). In conclusion, MEK-targeting strategies are particularly relevant for developing effective combination therapies in MPNST treatment (196).

### Targeting insulin growth factor receptor in non-GIST STS

The IGF/IGFR pathway is mainly implicated in the pathogenesis of some subtypes of bone and STS, such as osteosarcoma, RMS, and SS. An inhibitor of IGFR type-1 (IGF-1R) was assessed in a phase II trial and showed limited activity in refractory bone and STS. However, patients with RMS and osteosarcoma seem to have a clinical benefit from this drug (197). Given the disappointing findings with IGFR inhibitors as single agents, combinations should be considered to increase the antitumor effects. Translational data highlight the potential synergistic effect of dual targeting of IGFR and CDK4/6 in Ewing sarcoma (198). Exploration of this combination in the clinical setting is required. Similarly, preclinical data on Ewing sarcoma indicate the ability of combinatorial approaches with anti-IGF1R to promote antitumor activity when associated with mTOR inhibitors or chemotherapy (trabectedin) (199).

### Kirsten rat sarcoma viral oncogene homolog inhibitors

The major trigger for the development of most cancer types is ERK cascade activation due to pathogenic mutations, particularly in RAS (200). KRAS is one of the most frequently mutated oncogenes in solid tumors, including STS. For many years, KRAS was considered an undruggable target; however, recent advancements have led to the development of selective inhibitors for KRAS G12C mutations, making it possible to target mutant KRAS cancers (201). Adargrasib (MRTX849) and sotorasib (AMG510) are two potent and highly selective KRAS G12C inhibitors (202). These two agents are currently under investigation in ongoing phase I/II clinical trials as single agents or in combination with other therapies for patients with KRAS G12C-mutant advanced solid tumors (NCT06024174 and NCT04185883). However, it is important to note that the KRAS G12C mutation was detected in only a small number of patients with KRAS mutations. Furthermore, data from both preclinical and clinical studies have demonstrated that several mechanisms of acquired resistance to anti-KRAS G12C monotherapies have been described, leading to the activation of alternative RAS-dependent pathways (203). This suggests that combination therapy is an important and prioritized strategy to increase the efficacy and delay the acquisition of drug resistance (204). Additionally, combination approaches and novel alternative targeting methods for RAS-driven cancers are still under clinical investigation (205, 206). Two main strategies for overcoming resistance mechanisms have been explored: vertical inhibition of multiple nodes in the RAS pathway and horizontal inhibition of parallel pathways (207).

### MET inhibitors

The downstream response to c-MET activation directly promotes the activation of the RAS and MAPK cascades (208). Interestingly, it was recently shown that the HGF/c-MET signaling axis is implicated in tumor proliferation and metastatic spreading of cancer cells (209). One of the main downstream targets of the c-MET signaling axis is the MAPK: p38 and ERK1/2 pathways (210). A high expression of c-MET was observed in microphthalmia transcription factor-associated (MiT) tumors, including some STS subtypes (CCS and ASPS) (211). Targeting the MET axis is an attractive therapeutic option for these challenging histotypes. Preclinical findings suggest potential synergistic efficacy of a combination of HGF-targeted neutralizing antibodies with CAR-T cell treatments in Ewing sarcoma models (212). Other than multi-targeted TKIs with an activity on HGF/MET interactions, such as cabozantinib and crizotinib, new MET inhibitors are currently explored alone or in combination with EGFR and/or VEGFR inhibitors, particularly in MiT tumors (213).

### Other TKIs

Molecular biology studies have improved our understanding of bone and STS pathogenesis, leading to the successful use of novel targeted drugs (214). Various growth factor signaling pathways, such as FGFR, RET, and CSF1R, have been identified in bone and STS (215, 216). Targeting of these oncogenic factors is an interesting therapeutic approach. Based on these data, several clinical trials are ongoing to investigate novel targeted agents, such as CPL304110 (FGFR inhibitor), selpercatinib (RET inhibitor), and pexidartinib (CSF-1R inhibitor) (**Table 5**).

### Era of precision medicine

Recent advances in precision medicine and machine learning-based methods may therefore evolve the management of STS and respond to the urgent need to identify therapeutically targetable genomic and transcriptomic alterations to guide treatment and improve the clinical outcomes of these inherently challenging neoplasms (217, 218). To date, a multicenter, retrospective/prospective, translational study (PROGEN_SARC, NCT06076070) is ongoing to assess the feasibility of genomic profiling for therapeutic purposes in advanced or metastatic sarcomas after evaluation by the molecular tumor board. The results of this study are expected in May 2024. Another interesting randomized, multicenter, phase III trial (MULTISARC, NCT03784014) is ongoing to investigate the feasibility of using molecular profiling by NGS-exome in clinical practice for patients with advanced STS. In parallel, this trial evaluates the efficacy of targeted therapies recommended based on NGS analysis. The treatment strategy guided by NGS should be part of a list of 10 predefined therapeutic target inhibitors (nilotinib, ceritinib, capmatinib, lapatinib, trametinib, trametinib and dabrafenib, olaparib and durvalumab, palbociclib, glasdegib, TAS-120). The study is expected to be completed by October 2025.

## Emerging nanotechnology strategies in sarcomas

Nanomedicine is a novel multidisciplinary field that is garnering significant interest and investigation. It is an emerging area experiencing rapid development and is considered a promising strategy for the diagnosis and treatment of cancer (219). Advancements in nanotechnology are enabling responses to tumor microenvironment (TME) changes or external stimuli, thereby improving precise drug release. This innovation significantly enhances targeting specificity and reduces adverse effects of cancer treatment (220).

### Nanoparticles: a rising star for therapeutics and drug delivery in sarcomas

Despite significant advancements in tumor treatment, drug resistance and severe toxic side effects remain major challenges for clinicians in clinical practice. Nanoparticles (NPs) have revolutionized the diagnosis and treatment of cancers, addressing the limitations of conventional therapies by enhancing drug retention and permeability at tumor sites (221). NPs are categorized into two main classes: organic and inorganic. NPs are extensively applied in tissue engineering, tissue regeneration, drug-controlled release, and tumor immunotherapy due to their excellent biodegradability, large surface area, low cytotoxicity, and ease of modification (222). They provide advanced drug delivery capabilities, improving overall treatment efficacy through loading, targeting, and controlled release mechanisms, and overcoming the constraints of traditional methods.

Recently, NPs have been developed and tested for osteosarcoma, demonstrating potential applications in the diagnosis and treatment. Despite substantial progress in laboratory research, the clinical translation of nanomedicine still necessitates additional clinical trials and safety assessments (223).

### Extracellular vesicles as a next-generation drug delivery platform

Extracellular vesicles (EVs) contain proteins, RNA, genomic DNA, non-coding RNAs, lipids, and metabolites. They are categorized into three types: exosomes, microvesicles, and apoptotic vesicles (224). EVs facilitate information transfer between cancer cells, immune cells, and the TME, eliciting functional responses in receptor cells, promoting phenotypic changes, and influencing their physiological state. They are implicated in processes such as antigen presentation, cell proliferation, metastasis, angiogenesis, inflammation, and apoptosis (225).

Noncoding RNAs (ncRNAs) in EVs from tumor cells can enhance tumor angiogenesis, immune evasion, metastasis, and drug resistance (226). Circular RNAs (circRNAs), a type of ncRNA with covalently closed-loop structures, regulate tumor cell proliferation, invasion, and apoptosis by modulating various genes and signaling pathways. CircRNAs, enriched in exosomes, play roles in tumorigenesis and chemoresistance, including the regulation of cisplatin sensitivity in osteosarcoma cells (227).

In summary, EV-based drug delivery demonstrates significant potential as an advanced nanomaterials for drug delivery and treatment. Their capacity to deliver biologically active substances to target cells renders EVs as promising natural nanocarriers for treating bone sarcomas (228).

## Conclusion and future directions

The MAPK signaling pathway has a pivotal role in STS oncogenesis, acting as the foremost orchestrator of tumor cell responses to a diverse array of stimuli. This further opens the door to various components within this complex pathway to be identified as promising therapeutic targets for drug development. The growing focus on molecular therapies that inhibit the MAPK cascade signifies a substantial advancement in STS management. However, most current pharmaceuticals are not specific to MAPK nodes but rather kinase inhibitors or monoclonal antibodies that indirectly affect ERK/p38 activity. With numerous trials still in progress, it remains challenging to draw definitive conclusions regarding the role of MAPK in STS.

KRAS and MEK inhibitors offer more selective MAPK inhibition. Targeting KRAS is particularly promising due to the high frequency of KRAS mutations in non-GIST STS. Although substantial progress is anticipated in treating KRAS-mutant tumors, further research is necessary to elucidate resistance mechanisms and develop potential combination therapies. MEK inhibitors demonstrate potential in combination approaches for MPNST treatment, requiring clinical validation. These findings underscore the critical need to identify genetic and clinical indicators of response, resistance, toxicity, and optimal combination strategies for MAPK-targeted therapies in STS. Given the heterogeneous nature of STS pathology and clinical progression, single-agent targeted therapy has not yet demonstrated efficacy due to drug resistance. Vertical strategies targeting multiple MAPK pathway nodes may achieve more profound suppression. However, translational analysis is particularly challenging due to the high heterogeneity and rarity of non-GIST STS.

Recent advancements in genomic and epigenomic analyses of STS have facilitated the identification of novel alterations causally linked to the disease’s development. These alterations may be co-targeted with the MAPK axis in combination strategies to enhance therapeutic efficacy. Insights emphasize the next level of combined treatment in the era of precision medicine. The multi-targeted approach is used to prevent the emergence of resistance due to the activation of compensatory pathways associated with MAPK. This approach may become the cornerstone of new treatment combinations. To increase the likelihood of success, it is imperative to continue exploring diverse methodologies to further characterize STS at the molecular level, accelerate target discovery, and identify potential biomarkers. Additionally, the development of novel nanomaterials presents a promising avenue that may lead to breakthroughs in clinical practice.
